# Competence and Quality in Real-Life Decision Making

**DOI:** 10.1371/journal.pone.0142178

**Published:** 2015-11-06

**Authors:** Martin Geisler, Carl Martin Allwood

**Affiliations:** Department of Psychology, University of Gothenburg, Gothenburg, Sweden; Institut Pluridisciplinaire Hubert Curien, FRANCE

## Abstract

What distinguishes a competent decision maker and how should the issue of decision quality be approached in a real-life context? These questions were explored in three studies. In Study 1, using a web-based questionnaire and targeting a community sample, we investigated the relationships between objective and subjective indicators of real-life decision-making success. In Study 2 and 3, targeting two different samples of professionals, we explored if the prevalent cognitively oriented definition of decision-making competence could be beneficially expanded by adding aspects of competence in terms of social skills and time-approach. The predictive power for each of these three aspects of decision-making competence was explored for different indicators of real-life decision-making success. Overall, our results suggest that research on decision-making competence would benefit by expanding the definition of competence, by including decision-related abilities in terms of social skills and time-approach. Finally, the results also indicate that individual differences in real-life decision-making success profitably can be approached and measured by different criteria.

## Introduction

People constantly make decisions in their lives. Although decisions vary in importance, being able to make good decisions is important. Moreover, everyday decision-making outcomes have consequences that can be evaluated objectively as well as subjectively. But, what properties distinguish a competent decision-maker and, related to this, how can decision-making outcomes of direct relevance for peoples’ everyday lives be approached and measured?

Lately, research has begun to explore why some people tend to make better decisions than others (see e.g., [1–6]). The present research builds on the insights provided by this research on *decision-making competence* and explored three important questions. First, how are different (objective and subjective) criteria of real-life decision-making outcome related? We argue that decision-making competence cannot be defined without simultaneously outlining adequate criteria for decision quality, an issue that is unsettled and needs further attention [7–12]. In Study 1, using a community sample, this question was explored.

Second, does the predictive validity of a, mostly cognitively oriented, definition of decision-making competence [2], extend to subjective criteria of real-life decision-making outcome? Third, can predictions of real-life decision-making outcome profit by adding decision-related social skills and time-approach to the cognitively oriented definition of competence? The last two questions were investigated in Study 2 (police investigators) and 3 (social workers).

### Research on decision-making competence

Bruine de Bruin, Parker and Fischhoff [2] (see also [1]), collected different conventional decision-making tasks into a joint measure, the Adult Decision-Making Competence scale (the A-DMC henceforth called DMC). The DMC comprises six components: *resistance to framing*; *applying decision rules*; *under/overconfidence*; *consistency in risk perception*; *resistance to sunk costs*; and *recognizing social norms*. These components were selected in order to measure abilities of a normatively rational decision maker (i.e., a decision-maker that is able to resist cognitive biases) [1–2, 8]. Research has shown that DMC-performance relates to the extent individuals avoid negative decision-making outcomes [2], decision-making styles [13], decision-making ability in high-level leaders [5], and to cognitive ability and executive functions [4]. Additionally, DMC-performance seems to capture abilities relevant for financial planning [14], school performance [15] and interpersonal strengths [6] among pre-adolescents.

However, recent research has reported that both personality [16] and motivation [17] are important factors to consider in order to understand decision-making competence and real-life decision-making outcomes. The present research contributes to this development and extended understanding of decision-making competence by exploring the importance of other factors.

### Decision-making outcome and decision quality

The issue of decision quality is a core-aspect of decision-making science, but has often been overlooked or oversimplified [11]. Although decision makers’ preferences often are unstable [18–19] or unknown [20], it is assumed that, on average and in the long run, good decision-making processes tend to lead to good decisions [9]. Still, evaluating the preceding assumption requires knowing what constitutes good decision-making outcomes.

Research has reported that individuals often stress the outcome of decisions when evaluating decision quality [12]. However, these evaluations are also affected by how outcomes are reached [7, 21] and it has been proposed that evaluations of decision quality should attend to decision processes, not the outcome [22]. However, the basic rationale for evaluating decision quality without considering decision-makers’ personal goals and standards has been questioned [23–24]. Furthermore, subjective consequences of decisions have been proposed to be the standard by which evaluations of decision quality should be assessed [25]. That is, by exploring how decision-consequences are experienced and how decision processes conform to decision makers’ overall life [25] (see also [26]). Additionally, due to constrains given by uncertainty, it has been suggested that the best decision strategy is not to maximize utility but to maximize satisfaction [10]. Moreover, real-life decision quality may depend on the extent that individuals are able to justify decisions to themselves and/or others, i.e., on accountability [27–28]. As made evident by this selected review, decision quality may be best evaluated by using different types of criteria. Next we present three indicators of decision quality.

### Indicators of decision quality

#### The Decision Outcome Inventory

Bruine de Bruin et al. [2] introduced a self-report measure intended to assess individual differences in experiences of real-world decision-making outcomes: the *Decision Outcomes Inventory* (DOI) which they used to test the external validity of DMC. The DOI consists of 41 decision outcomes with 34 item-pairs (some pairs consist of more than one possible negative outcome) and some single items. Item-pairs first ask if one has made a decision (e.g. *gone shopping for food or groceries*/*been married*), and then asks if one has experienced a negative outcome of that decision (e.g. *threw out food or groceries you had bought because they went bad*/*been divorced*). The single items does not have a preceding screening question and simply asks if one has experienced certain outcomes assumed to be associated with real-life decision making (e.g. been *declared bankruptcy* or *been diagnosed with type 2 diabetes*). DOI-scores are calculated by weighting each negative outcome by the proportion of participants who report to not have experienced the negative outcome (e.g. *been divorced*), although they had the possibility (e.g. *been married*). Thus, the DOI-score is based on the assumption that less frequent outcomes (in the sample studied) are more severe than the more frequent ones. Undoubtedly, the DOI captures aspects of everyday decision-making success. Nevertheless, the DOI could be considered to be a restricted measure. To illustrate, the DOI attends to self-reported outcomes that are evaluated objectively, but does not consider the possible reasons behind the decisions or decision makers’ subjective evaluations of these decision outcomes. Therefore, research referring to the benefits of the DMC as evaluated by the DOI (e.g., [1–5]) may oversimplify the relationship between DMC-performance and real-life decision-making outcomes. Since DMC-research provides a much needed perspective within decision-making science, it is important to also investigate how scores on the DOI are related to other outcome measures. In addition, such investigations offer a basis for further explorations of the relationship between DMC-performance and outcomes of peoples’ real-life decision making. Previous research has reported that DOI-scores are negatively related to maximizing tendencies [2, 13], and that both DOI-scores and maximizing tendencies, in turn, are related to regret proneness and depression [29]. The present research explored the relationship between DOI-scores and subjective outcomes in more detail. Specifically, we explored how DOI-scores relate to subjective indicators of real-life decision-making outcomes in terms of general satisfaction with life and experiences of minor everyday difficulties. Next, we discuss why these measures can be expected to indicate decision-making outcome in real-life.

#### Satisfaction with life

To evaluate real-life decision-making outcomes by reported satisfaction with life means to assess individuals’ subjective evaluations of their general decision-making success based on their personal goals and standards. Reports of satisfaction with life have been proposed to be based on assessments in which perceived circumstances are compared to personal and self-imposed standards [30]. Thus, these reports may reflect how well decisions have conformed to personal goals and standards [23–25]. However, multiple factors influence subjective well-being, and personal goals and standards differ. Therefore, it has been recommended to measure satisfaction with life globally [31]. That is, global measures of satisfaction with life do not have pre-defined domains. Instead, domains are open for subjective inference, thereby reflecting personal evaluations of general goal-fulfilment.

#### Experiencing minor difficulties in life: “daily hassles”

Experiencing everyday difficulties has been proposed to constitute a possible consequence for individuals with lower decision-making abilities [2] and can be seen as reflecting the ability to perform and execute real-life decision-making processes efficiently. Individual differences in such experiences are measured by *the Survey of Recent Life Experiences*, SRLE [32]. Although self-reported experiences of everyday difficulties resemble the design of the DOI, it broadens the definition of decision quality by emphasizing the implementation of everyday decision-making processes. Additionally, and importantly, whereas the DOI measure if events have *occurred* in a person’s life the SRLE measures the extent that individuals’ have *experienced* problems or concerns.

### The social dimension of decision-making competence

The social dimension of many decisions has been noted by previous research [27, 33–34]. Moreover, day-to-day decision making sometimes entails a conflict between reason and emotion [35] since many decisions require self-control and emotion regulation in order to be successful [36]. Given this, certain social skills can be assumed to be fundamental in order to make competent decisions. In the present research we explored this assumption, focusing on two aspects of such skills: self-awareness and emotional intelligence.

#### Self-awareness

Being able to recognize social cues and adapt self-presentation accordingly can be expected to aid decision making in social everyday settings. The social psychological phenomenon *self-monitoring* includes these abilities [37–39]. Numerous studies have demonstrated the general benefits associated with self-monitoring ability, for example, with respect to job performance [40] and successful handling of social-exchange relations [41]. Furthermore, it has been argued that self-presentation ability is a skill (i.e. some people are better at it and it can be improved through practice) essential in order to be successful in various domains of life [42]. Hence, we included individual differences in self-monitoring when exploring factors that contribute to real-life decision-making success.

#### Emotional intelligence

Emotions influence people’s decision making [43–44]. Researchers have proposed that investigations of this influence should attend to individual differences in trait emotional intelligence [TEI], “…since it provides comprehensive coverage of emotion-related self-perceptions that are directly relevant to the study of affective decision making” [45] (p. 1356). The TEI-scale measures basic emotional-disposition and self-perceived ability to correctly understand emotional reactions in self and others, as well as ability in emotion regulation and emotion communication [46–47]. Previous research has reported positive relationships between higher TEI-scores and successful decision making in social contexts [48–49].

### Time-approach as a part of decision-making competence

Time is an important aspect of decision making [50]. More specifically, individual differences in perception of, and approach to, time have been reported important to consider in order to understand how people make decisions and evaluate outcomes [51]. The effect of time on decision-making has been demonstrated in terms of delay of gratification, i.e., sacrificing short-term happiness in favour of long-term well-being [52]. However, it has been suggested that the study of individuals’ approach to time needs to be more differentiated [53]. In this research, we focused on two aspects of individual differences in time-approach: general perceptions of time and attitudes to time and time-related activities (i.e., time styles) and global procrastination tendencies.

#### Time-approach

Research has demonstrated how individual differences in aspects of time-approach influence decision making. These differences have been explored in terms of self-regulation [52], time-perspective [54], and time styles [55]. However, these different terms and definitions have been suggested to be largely interchangeable and synonymous [56]. For example, this research shows that individual differences in time-approach are related to the way decisions are approached [57] and affect consumer decisions [55].

#### Procrastination

Procrastination, i.e., inclination to postpone commencement or completion of tasks [58], has been explained in various ways [59]. Furthermore, although procrastination behaviour has been related to short-term benefits, procrastination generally leads to impaired performance and long-term costs [60]. Recent research suggests that time-approach and procrastination tendencies are important to consider in personnel selection since they guide individuals’ personal and professional judgements and decisions [61]. In sum, individual differences in approach to time are important to attend to when exploring factors relating to real-life decision-making success.

## The Present Research

This research investigated three questions. The first concerned the relation between objective and subjective indicators of real-life decision outcome. Specifically: How does an objective measure of individual differences in real-life decision-making outcome (i.e., the *Decision Outcome Inventory*, *DOI*) relate to subjective measures of real-life decision-making outcomes (i.e. the *Satisfaction With Life Scale*, SWLS, and the *Survey of Recent Life Experience*, SRLE)? This question was primarily explored in Study 1, yet complementary expanded in Study 3. Investigating these relations contributes to the basic understanding and conceptual validity of the DOI, and offers a basis to further define the predictive validity of the DMC. In order to better understand the predictive validity of DMC, our second research question was: Will the previously reported relation between DMC-performance and real-life decision-making outcome replicate when different criteria (i.e. subjective indicators) of quality/outcome are used? Acknowledging the social dimension and complexity in real-life decision making, our third research question was: Can individual differences in decision-related a) social skills and b) time-approach add to the explanation of variance in real-life decision-making outcome(s) beyond the variance explained by the DMC? These two questions were explored using participants with an explicit requirement to make good decisions: police investigators (Study 2) and social workers (Study 3).

## Study 1

### Method

#### Ethics statement

This research (Study 1–3) has been approved by the Regional Ethical Review Board, Gothenburg secretariat; www.epn.se, 2011-02-21, dnr: 071–11. Written informed consent was obtained from participants in all studies (Study 1–3) reported.

#### Aim

A community sample study was performed to investigate how an existing definition and measure of individual differences in (objective) real-life decision-making outcomes (i.e., the *Decision-making Outcome Inventory*; DOI) relates to other (subjective) indicators of real-life decision-making outcomes (i.e., *satisfaction with life* and *experiences of daily hassles*).

#### Participants

To obtain a sample representative of the Swedish community, the study was performed through an online survey company (www.cint.com). In all, 217 individual invitations were sent out. Of these invitations, 18 individuals declined the opportunity to participate and an additional 24 individuals did not answer the complete set of scales/measures. Hence, the response-rate was 100% and the participation rate 81%. The 24 participants that did not complete the study in full did not differ from the sample on which the reported analyzes were based. The final sample of 175 participants was considered reasonably representative for the Swedish community in terms of *gender* (54% women), *age* (*Min* = 18, *Max* = 76, *M* = 46.58, *SD* = 15.89), *educational background* (7.4% lower secondary education, 41.1% high school education, and 51.4% college education), *occupation* (15% students, 54% gainfully employed, 5% unemployed, 21% retired, 5% assigned their current occupation as “other”), and *native language* (91% reported Swedish). This sample was also differentiated geographically (including both metropolitan and rural areas).

#### Materials

Study 1 used the following scales: *The Decision-making Outcome Inventory* (DOI), *Satisfaction With Life Scale* (SWLS), and *Survey of Recent Life Experience* (SRLE). Scales not available in Swedish (i.e., the DOI and the SRLE) were translated following a conventional back-translation procedure.

***The Decision-making Outcome Inventory (DOI):*** The DOI [2] measures individual differences in being able to avoid negative outcomes of real-life decision making and is a self-report measure that collects 41 negative outcomes from different domains of life. For 35 outcomes, participants are initially asked if they have made decisions (e.g., *been married*) allowing for negative outcomes to be possible (e.g., *been divorced*). The remaining six outcomes (e.g., *spent a night in a jail cell for any reason*; *been diagnosed with Type 2 diabetes*, etc.) are not preceded by an initial question since these outcomes are reached by associated decisions. The overall DOI score is calculated by weighting each negative outcome a respondent has experienced by the proportion of participants who have not experienced it.

***Satisfaction With Life Scale (SWLS):*** The SWLS’s [31] five items consist of context-free statements of general life satisfaction, rated on 7-point Likert scales. The SWLS measures aspects of overall goal-fulfilment in life based on personal standards. An item example is “*So far*, *I have gotten the important things I want in life*.”

***Survey of Recent Life Experiences (SRLE)–short form:*** The SRLE [32] short form has 41 items, rated on 4-point Likert scales. The short form is reliable (α = .90, compared to α = .92 for the original 51-item version; p. 227 [32]) and was used in the present study to reduce participants’ workload. SRLE items ask if and to what extent specific hassles have been part of a person’s life in the past month. The SRLE provides a total score but has a multi-factor structure that includes different contexts of daily hassles: *social and cultural difficulties* (conflicts with friends and family), *work* (dissatisfied with work or experiencing lower evaluations of work efforts than expected), *time pressure* (experiencing incongruence between demand and performance due to obligation overload), *finances* (economic troubles), *social acceptability* (physical dissatisfaction with self or social rejection), and *social victimization* (experiencing that one is being taken for granted). An item example is “*Struggling to meet your own standards of performance and accomplishment*” (item on the *time pressure* facet).

#### Procedure

Participants were contacted by email. After giving informed consent, participants completed the questionnaire individually. The completion time was approximately 10–15 minutes.

### Results

#### Descriptive statistics

Descriptive statistics are presented in Table 1. Compared with previous research, the present community sample reported moderate levels of satisfaction with life [30] but somewhat lower levels of daily hassles [32]. Additionally, the sample reported an overall higher ability to avoid negative consequences associated with real-life decision making [2].

**Table 1:** Descriptive statistics—Study 1 (*N* = 175).

	M (SD)	α	Possible Range	Observed Range
DOI	-0.07 (0.07)	.76	-1.00–0.00	-0.36–0.00
SWLS	4.75 (1.32)	.92	1–7	1–7
SRLE	65.23 (16.63)	.93	41–164	41–142

*Note*: DOI—Decision Outcome Inventory, SWLS—Satisfaction With Life Scale, SRLE—Survey of Recent Life Experience

#### Correlations

Table 2 presents the correlations. No significant correlations were found between gender and the included measures. However, significant relationships were found for age and all included measures, where higher age consistently had more favourable results. No significant correlation was found between the DOI and subjective well-being (SWLS). However, in line with our expectations, significant (negative) correlations were obtained between the DOI and reports of experiencing daily hassles (SRLE). This finding suggests that individuals’ ability to avoid (objectively defined) negative outcomes of real-life decisions is related to the degree that one (subjectively) experiences minor everyday difficulties and frictions (daily hassles). Finally, reports of experiencing daily hassles showed a negative correlation to reports of subjective well-being.

**Table 2:** Correlations—Study 1.

	1	2	3	4	5
1. Gender	-				
2. Age	.100	-			
3. DOI	-.081	.292**	-		
4. SWLS	-.017	.189*	.125	-	
5. SRLE	-.082	-.321**	-.311**	-.403**	-

*Note*: DOI—Decision Outcome Inventory, SWLS—Satisfaction With Life Scale, SRLE—Survey of Recent Life Experience

* *p* < .05.

** *p* < .01.

### Discussion

The results showed that DOI-scores were related to (less) reports of daily hassles (SRLE), but not to reported levels of subjective well-being (SWLS). However, in some contrast, SRLE was related to SWLS. One possible explanation for this pattern of results could be that general satisfaction with life is not directly related to successful decision making [62–63], at least not as evaluated objectively. Instead, general satisfaction with life may be more dependent on individuals’ general approach to life (optimism/pessimism) and personality [64–65]. However, this conclusion would be a rather fatalistic view and ultimately even question the very purpose and relevance of research on individual decision making in general. Alternative explanations may profitably be based on the assumption that evaluations of decision quality need to be extended to, and understood within, the social context [27, 33, 36] as well as complemented by **quality-evaluations made in relation to decision-makers**’ **own understanding, personal standards and subjective experiences** [23–25]. In sum, this topic needs further exploration and the research-field dedicated to decision-making competence provides a suitable frame for this endeavour.

## Study 2

### Method

#### Aim

Study 2 explored how different aspects of decision-making competence relate to and hold predictive validity for subjective indicators of decision-making outcome (SWLS and SRLE).

#### Participants

In total, 360 police investigators were invited. Invitations were randomized to obtain a representative and differentiated sample both geographically (metropolitan and rural areas) and in terms of alignment (i.e., investigators of violent crimes, traffic offenses, etc.).

The study was initially distributed by web-based questionnaires (sent out to 165 police investigators) and answered by 66 participants (participation rate = 40%). However, the questionnaires of 21 participants were not complete and were therefore excluded, leaving 45 participants. To facilitate participation, as the use of web-based questionnaires was problematic due to issues of restricted computer-access and non-activated email-addresses among the presumptive participants, an additional 195 invitations were sent out by paper-and-pen questionnaires. Here, 50 participants answered these questionnaires (participation rate = 26%, total participation rate = 32%). For the paper-and-pen questionnaire, 5 participants did not answer some of the scales and were excluded. Moreover, for the paper-and-pen questionnaires, there was a limited concern of missing. Missing data analysis showed no pattern; thus, data were considered to be missing at random and replaced by computations using the Expectation-Maximization method [66]. Accordingly, in the final sample of 90 participants (37% women, mean age = 46 years), 45 participants had answered the web-based questionnaire and 45 participants the paper-and-pen questionnaire.

#### Materials

Scales unavailable in Swedish were translated following a conventional back-translation procedure. The following scales (described further below) were used as indicators of decision making competence: *Adult Decision Making Competence scale* (DMC), *Self-Monitoring Scale* (SMS), *Trait Emotional Intelligence Questionnaire—Short Form* (TEIQue-SF), *Procrastination scale*, and *Time-Style Scale* (TSS).

***The Adult Decision Making Competence scale (DMC):*** The DMC [2] measures performance on six components argued to capture essential decision-making skills: *resistance to framing*, *applying decision rules*, *resistance to sunk costs*, *consistency in risk perception*, *under-/overconfidence*, and *recognizing social norms*. DMC performance is evaluated in terms of accuracy and/or internal consistency.

Because of cultural differences and to render the study appropriate for the police investigators, certain adjustments of the DMC were made. In the part *recognizing social norms* (RSN), 6 of the original 16 items were excluded because of their inappropriateness for a Swedish setting [67]. Pilot study results (N = 15, 10 women, mean age = 24.4 years) showed that this exclusion gave a higher reliability for this part (*α* = .73) than that (*α* = .64) reported by Bruine de Bruin et al. (2007), although one item lacked variation and was therefore also excluded. Additionally, three of the nine RSN items were amended because they asked about law violations more than not complying with social norms (e.g., smoking marijuana), which could have been considered odd for the police sample. Finally, based on the consideration of time restriction for the participants (, the DMC component *under-/overconfidence* was excluded [67]. At the time of the study, we were unaware of the existing Swedish translation of the DMC [68]. However, apart from the amendments described, no major differences were found between the translations.

***Self-Monitoring Scale (SMS):*** Originally developed by Snyder [39], the SMS measures individuals’ self-perceived sensitivity to acknowledging subtle hints from others and ability to modify self-presentation accordingly. We used the revised SMS [38] that includes 13 items rated on 6-point Likert scales. The SMS provides both a total score and scores on two subscales: *ability to modify self-presentation* and *sensitivity to expressive behaviour of others*. An item example is, “*In social situations*, *I have the ability to alter my behaviour if I feel that something else is called for”* (item on the *ability to modify self-presentation* subscale).

***Trait Emotional Intelligence Questionnaire—Short Form (TEIQue-SF):*** Trait emotional intelligence (TEI) measures self-reported disposition and self-perceived ability to regulate, communicate, and influence emotional reactions in both self and others. TEI was considered suitable in the present research since it relates to personality whereas ability-emotional intelligence (measured by maximum performance) relates to aspects of cognitive ability. The TEIQue is favourable among the different measures of TEI in terms of validity and reliability [69]. Petrides and Furnham [70] developed the TEIQue-SF, based on the full version of the TEIQue [46], consisting of 30 items (the two items, of the total 153 items, of TEIQue’s 15 subscales with the highest correlation with the respective total subscale score) rated on 7-point Likert scales and providing a global TEI-score. We used the short form to reduce participants’ workload. Item example: “*Expressing my emotions with words is not a problem for me*.”

***Procrastination scale:*** Procrastination tendencies reflect the extent that commencement or completion of necessary activities is postponed [59]. The Procrastination scale’s [58] 20 items are rated on 5-point Likert scales. An item example is, “*I do not do assignments until just before they are to be handed in*.”

***Time-Style Scale (TSS):*** The TSS [55] collates previous time research into a single measurement and predicts activities related to time and planning, e.g., consumer decision making. Its 29 items are rated on 7-point Likert scales and include eight different time-styles: *preference for economic time* (i.e., prefer to attend to tasks in an organized way); *preference for non-organized time* (preference for dealing with multiple tasks simultaneously or in a less structured way); *orientation towards the past*; *orientation towards the future*; *time submissiveness* (having dutiful and conforming attitudes to time-related activities); *time anxiety* (experiencing adjustment troubles and anxiety when activities are related to time); *tenacity* (i.e., delay of gratification); and *preference for quick return* (being impatient and having a more restricted time horizon). An item example is, “*I sometimes feel that the way I fill my time has little use or value*” (*time anxiety* time-style).

In addition, as measures of real-life decision-making outcome, Study 2 used the *Satisfaction With Life Scale (SWLS)* [31] and the *Survey of Recent Life Experiences—short form (SRLE)* [32]. These two measures are described in the method section of Study 1. Due to time constraints the *Decision-making Outcome Inventory* (DOI) [2] was not used in this study.

#### Procedure

Web-based questionnaires were administered by e-mail. Participants gave informed consent and completed the web questionnaires individually. Paper-and-pen questionnaires were accompanied by a stamped, addressed envelope.

### Results

#### Preliminary analyses and descriptive statistics

Descriptive statistics are presented in Table 3. Overall, descriptive statistics of the DMC were in line with previous research [2] but indicated generally higher means and restrictions in range. The current sample specifically showed a somewhat higher mean and restriction of range on the part *resistance to sunk costs*. With regard to social skills, the current sample reported moderate levels of *Self-monitoring*, in particular for the subscale *ability to modify self-presentation*, whereas descriptive statistics of the TEIQue-SF indicated overall high reports of this disposition. For *procrastination*, overall low tendencies were observed for the present sample. For the *Time-styles*, the *submissive* and *tenacity* styles were the most salient for the current sample. Finally, mean levels on the outcome measures *satisfaction with life* and *experiences of daily hassles* were in line with those in the community sample of Study 1.

**Table 3:** Descriptive statistics—Study 2 (N = 90) and Study 3 (N = 111).

	Study 2	Study 3	Study 2	Study 3		Study 2	Study 3
Component	M (SD)	M (SD)	α^a^	α^a^	Possible Range	Observed Range	Observed Range
***DMC***							
Resistance to framing	3.94 (0.45)	4.05 (0.45)	.56	.55	0.00–5.00	2.64–4.79	2.00–4.79
Applying decision rules	0.64 (0.20)	0.63 (.19)	.74	.61	0.00–1.00	0.20–1.00	0.10–1.00
Consistency in risk perception	0.83 (0.10)	0.85 (0.9)	.55	.41	0.00–1.00	0.50–1.00	0.65–1.00
Resistance to sunk costs	4.79 (0.63)	4.41 (0.63)	.53	.47	0.00–6.00	3.50–6.00	2.60–5.80
Recognizing social norms	0.39 (0.31)	0.42 (.27)	.55	.73	-1.00–1.00	-.66–0.90	-.57–.90
***Social skills***							
Self-Monitoring (Total)	52.87 (6.90)	50.61 (5.60)	.83	.82	13–78	37–68	38–63
- Ability to modify self-presentation	27.66 (4.43)	26.00 (2.85)	.74	.77	7–42	17–35	19–32
- Sensitivity to expressive behaviour of others	25.20 (4.07)	24.62 (3.51)	.80	.67	6–36	14–35	18–32
Trait Emotional Intelligence	156.58 (18.76)	161.46 (18.67)	.84	.88	30–210	115–204	112–200
***Procrastination behaviour***							
Procrastination	43.36 (10.68)	49.55 (11.08)	.82	.86	20–100	25–74	20–76
***Time-Style***							
Preference for economic time	16.92 (5.18)	17.48 (4.38)	.82	.78	4–28	4–28	5–27
Preference for non-organized time^b^	9.72 (3.75)	9.28 (3.34)	.73	.73	2–21	3–21	3–21
Orientation towards the past	12.43 (5.05)	11.63 (4.69)	.84	.81	4–28	4–25	4–26
Orientation towards the future	19.70 (5.45)	18.82 (5.05)	.81	.89	4–28	4–28	4–28
Time submissiveness	23.66 (4.51)	21.67 (5.23)	.48	.78	4–28	9–28	8–28
Time anxiety	11.92 (4.72)	11.05 (3.96)	.73	.69	4–28	4–24	4–21
Tenacity	15.49 (3.54)	14.72 (3.40)	.71	.78	3–21	3–21	6–21
Preference for quick return	13.18 (3.86)	12.47 (3.19)	.84	.87	3–21	3–20	5–21
***Outcome***							
Survey of recent life experience	67.10 (15.09)	68.95 (15.63)	.88	.92	41–164	42–121	42–112
Satisfaction with life	4.81 (1.22)	4.99 (1.05)	.89	.84	1–7	2–7	2–7
Decision Outcome Inventory	-	-0.09 (0.07)	-	.74^c^	-1.00–0.00	-	-0.35–0.00

*Note*. The presented scores represent the mean score for all scales and subscales.

^a^ Cronbach’s alpha: Reliability is indicated by Cronbach’s alpha (α).

^b^ One item on this time-style was missing in study 1

^c^ Decision Outcome Inventory: Three outcomes showed no variance and were therefore excluded from the alpha calculation

#### Correlations

Table 4 presents the correlations. Significant relationships were found between the outcome measures and measures of social skills, procrastination, and certain time styles. Looking at the correlations for the DMC, only one significant correlation was found: a negative relation between the component *consistency in risk perception* and the outcome measure *daily hassles* (SRLE). Moreover, the TEIQue-SF was significantly related to both outcome measures. Interestingly, scores on the TEIQue-SF were positively correlated with the DMC component *resistance to sunk costs*.

**Table 4:** Correlations—Study 2.

	1	2	3	4	5	6	7	8	9	10	11	12	13	14	15	16	17	18	19	20
1. RTF	-																			
2. ADR	.314**	-																		
3. CRP	.104	.189	-																	
4. RTSC	-.156	.067	-.026	-																
5. RSN	.074	.094	.095	.131	-															
6. SMS-Total	.060	-.073	-.173	-.034	-.162	-														
7. SMS-Ability	.030	.032	-.137	.085	-.174	.832**	-													
8. SMS-Sensitivity	.070	-.160	-.145	-.151	-.087	.795**	.326**	-												
9. TEIQue-SF	-.200	-.154	.045	.236*	.071	.243*	.274*	.114	-											
10. Procrastination	.096	.077	-.050	-.264**	-.141	-.052	-.102	.022	-.423**	-										
11. Economic TS	-.096	-.050	-.186	-.011	-.131	.028	-.005	.054	-.019	-.423**	-									
12. Non-organized TS	.008	-.118	.048	.003	.099	-.054	-.097	.015	-.059	.294**	-.617**	-								
13. Past-oriented TS	-.041	-.081	-.208*	-.110	-.097	.124	.060	.146	-.322**	.104	.214*	-.071	-							
14. Future-oriented TS	.078	.055	-.118	-.229*	-.182	.216*	.158	.195	-.070	.034	.285**	-.302**	.403**	-						
15. Submissive TS	-.143	-.019	-.098	.158	-.017	.086	.163	-.032	.201	-.552**	.269*	-.253*	-.048	.077	-					
16. Anxious TS	.159	.151	-.215*	-.048	-.133	-.009	.002	-.018	-.514**	.175	.117	-.006	.427**	.201	-.045	-				
17. Tenacity TS	-.079	-.049	-.117	.126	.076	.057	-.011	.109	.263*	-.458**	.431**	-.412**	.081	.014	.329**	-.120	-			
18. Pref. quick return TS	-.139	-.176	-.063	-.071	-.060	.010	.033	-.020	-.095	.010	.240*	-.026	.016	-.038	-.006	.022	.003	-		
19. SWLS	-.048	-.018	.162	.113	.119	-.046	-.013	-.064	.532**	-.238*	.065	-.077	-.315**	-.087	.026	-.614**	.143	-.042	-	
20. SRLE	.033	-.116	-.226*	-.186	-.124	.205	.090	.250*	-.271*	.257*	-.078	.016	.313**	.344**	-.115	.369**	.034	.020	-.438**	-

*Note*. The presented significances are for Pearson’s correlation and two-tailed tests of significance.

RTF = Resistance to Framing, ADR = Applying Decision Rules, CRP = Consistency in Risk Perception, RSC = Resistance to Sunk Costs, RSN = Recognizing Social Norms, SMS = Self-Monitoring Scale, TEIQue-SF = Trait Emotional

Intelligence Questionnaire—Short Form, TS = Time-Style, SWLS = Satisfaction With Life Scale, SRLE = Survey of Recent Life Experience.

* *p* < .05.

** *p* < .01.

For time styles, the *past*-*oriented*, *future*-*oriented*, and *anxious* time styles had a significant relationship with the outcome measures. Only time styles with a significant association with the outcome measures were included in the regression analysis.

#### Multiple regression models

Hierarchical multiple regression analyses were performed to investigate the predictive relationship between the different decision-making competence factors and measures of real-life decision-making outcome (Table 5). Separate analyses were performed for the outcome measures *SWLS* and *SRLE*, as well as for the controlling factors and different blocks of predictors: 1) *gender* and *age*, 2) *DMC*, 3a) *Social skills*, and 3b) *Time-approach*, respectively. Hence, the predictive validity of Social skills and Time-approach were tested separately in block 3 of the regression model.

**Table 5:** Hierarchical regression of SWLS and SRLE—Study 2 (N = 90).

	Total *R* ^2^	Adjusted *R* ^2^	Δ*R* ^2^	Test of Δ*R* ^2^
SWLS				
- Step 1: Gender and age	.01	-.02	.01	*F*(2, 79) = .33, *p* = .718
- Step 2: DMC	.06	-.02	.06	*F*(5, 74) = .88, *p* = .495
- Step 3^a^: Social skills	.38	.30	.32	*F*(3, 71) = 12.17, *p* < .001
- Step 3^b^: Time-approach	.44	.36	.38	*F*(3, 71) = 15.99, *p* < .001
SRLE				
- Step 1: Gender and age	.16	.13	.16	*F*(2, 79) = 7.26, *p* = .001
- Step 2: DMC	.21	.13	.05	*F*(5, 74) = 1.00, *p* = .423
- Step 3^a^: Social skills	.40	.31	.19	*F*(3, 71) = 7.47, *p* < .001
- Step 3^b^: Time-approach	.37	.28	.16	*F*(3, 71) = 5.88, *p* < .001

*Note*. SWLS = Satisfaction With Life Scale; SRLE = Survey of Recent Life Experience; DMC = Decision Making Competence.

^a, b^ Separate blocks in Step 3

***Predicting satisfaction with life:*** As Table 5 shows, Step 1 (*gender* and *age*) explained 1% of the variability in SWLS (n.s.). In Step 2, the *DMC* added 6% to the explained variance (n.s.). When the block *Social skills* was added in Step 3 there was a substantial increase in explained variance of 32% (*p* < .001). The *TEIQue-SF* (*β* = .656, *p* < .001) was the significant predictor in the *Social skills* block. When instead measures of *Time-approach* were inserted into Step 3 instead, this block accounted for an additional 38% of the variance (*p* < .001). Here, the *anxious* time style was the significant predictor (*β* = -.631, p < .001).

***Predicting recent life experiences:*** Multiple regression analyses using daily hassles (SRLE) as the outcome measure showed that in Step 1, *gender* and *age* explained a substantial 16% of the outcome. In this block, *age* (*β* = -.414, *p* = .002) was the significant predictor (i.e., higher age = fewer experiences of daily hassles). In Step 2, the *DMC* added 5% of the variance explained (n.s.). Moreover, when the block *Social skills* was added to the model in Step 3, the explained variance increased by 19% (*p* < .001). Here, the *TEIQue-SF* (*β* = -.474, *p* < .001) was the significant predictor. However, the SMS subscale of “*sensitivity to expressive behaviour of others*” almost reached significance (*β* = .203, *p* = .058). Finally, using measures of *Time-approach* in Step 3 added a total of 16% of explained variance (*p* < .001). In this block, the *anxious* time-style (*β* = .346, *p* = .001) was the significant predictor.

### Discussion

The results showed that the predictive validity of the DMC, reported in previous research (see e.g., [1–2]), was not replicated when criteria for real-life decision-making outcome were measured subjectively in terms of reported *daily hassles* and *satisfaction with life*. Conversely, the results clearly illustrate that for the present criteria of decision quality (i.e. subjective criteria), individual differences in decision-making related aspects of social orientation and time-approach accounted for a significant amount of variance. Study 3 attempted to investigate these relations further in a different work life context. Furthermore, in order to better relate our results to previous DMC-research, study 3 also included an objective measure of real-life decision-making outcome: the DOI.

## Study 3

### Method

#### Aim

Based on the results of Study 1 and Study 2, we continued to explore the relationship between decision-making competence factors and measures of real-life decision-making outcome(s). A possible limitation of Study 2 was that the objective definition of real-life decision-making outcome used in previous DMC-research was not included (i.e., the DOI), this measure was incorporated in Study 3.

#### Participants

A total of 720 social workers were invited to participate in the study. All in all, 111 participants (85% women, mean age = 43 years) completed the survey in full (response rate = 15%) with the exception that 4 participants did not complete specific scales/subscales (Hence, N = 110 for DMC component applying decision rules and the DOI, and N = 109 for the SMS subscale ability to modify self-presentation).

#### Materials

Study 3 used the same material as Study 2, except that the *Decision Outcome Inventory* (DOI) [2], described in the method section for Study 1, was also included. However, to be suitable for the professional sample, some amendments of the DOI were necessary. Hence, the 6 items/outcomes of the DOI that asks about infidelity, diagnose of sexually transmitted disease, unplanned pregnancy, condom use, drunk driving and whether or not one has spent a night in a jail cell (for any reason) were excluded.

#### Procedure

Invitations to participate in the study were sent out by e-mail, preceded by information about the upcoming study given by regional managers at workplace meetings. A web-based survey was used. After signing informed consent, participants completed the questionnaire individually.

### Results

#### Descriptive statistics

Descriptive statistics for Study 3 are presented in Table 3. For the outcome measures, reported levels for the present sample were largely similar to those reported by the sample in both Study 1 and Study 2. Looking at overall performance on the DMC compared to the sample in Study 2, the present sample had a somewhat higher performance on the component *resistance to framing* (RTF) but slightly lower performance on *resistance to sunk costs* (RTSC). Regarding measures of social orientation, overall levels of the present sample were comparable to those in Study 2, but reports of TEIQue-SF were a bit higher. Finally, mean scores on measures of time-approach were in line with those of Study 2, but the present sample reported somewhat higher levels of procrastination behaviour.

#### Correlations

As indicated by the correlations shown in Table 6, the main relationships identified in both Study 1 and Study 2 were replicated in Study 3. Compared with Study 1, correlations between measures of real-life decision-making outcome were confirmed. However, for the present sample, a significant positive relationship was observed between satisfaction with life and reported ability to avoid negative consequences of real-life decisions, *r* (109) = .289, *p ˂* .01. Moreover, as in Study 2, interrelationships between DMC components reported in previous DMC research were not replicated. Furthermore, only some very weak correlations were observed between DMC components and measures of social orientation or time-approach. In addition, the non-significant relationships between the DMC and subjective indicators of real-life decision-making success (satisfaction with life; SWLS, and daily hassles; SRLE) found in Study 2 were replicated here. Interestingly, no significant relationship was observed between any of the single DMC components and the DOI.

**Table 6:** Correlations—Study 3.

	1	2	3	4	5	6	7	8	9	10	11	12	13	14	15	16	17	18	19	20	21
1. RTF	-																				
2. ADR	.213*	-																			
3. CRP	-.123	.107	-																		
4. RTSC	-.078	.025	.052	-																	
5. RSN	.056	-.067	.079	.024	-																
6. SMS-Total	.008	.122	-.030	.031	-.117	-															
7. SMS-Ability	.011	.137	.017	-.041	-.077	.877**	-														
8. SMS-Sensitivity	-.019	.095	-.055	.082	-.146	.886**	.555**	-													
9. TEIQue-SF	.079	.219*	-.062	.201*	-.083	.461**	.470**	.350**	-												
10. Procrastination	.048	.053	.066	-.125	-.029	-.129	-.153	-.039	-.488**	-											
11. Economic TS	-.027	.132	.013	-.112	-.141	.235*	.194*	.220*	.107	-.294**	-										
12. Non-organized TS	-.089	-.195*	-.062	.079	.008	-.188	-.270**	-.040	.221*	.556**	-.483**	-									
13. Past-oriented TS	-.049	-.060	.018	-.175	.061	-.130	-.164	-.070	-.477**	.293**	.173	.064	-								
14. Future-oriented TS	.040	.219*	.033	.009	.013	.169	.144	.152	-.189*	.188*	.252**	-.078	.412**	-							
15. Submissive TS	-.190*	-.089	-.027	.118	.082	.104	.061	.100	.172	-.557**	.221*	-.319**	-.086	-.015	-						
16. Anxious TS	-.072	-.013	.081	-.137	.060	-.267**	-.281**	-.183	-.656**	.391**	-.094	.160	.391**	.367**	-.118	-					
17. Tenacity TS	-.169	-.014	.032	-.028	.021	.152	.151	.123	.199*	-.432**	.326**	-.179	-.127	.071	.090	-.140	-				
18. Pref. quick return TS	-.200*	-.029	.113	.140	-.113	-.124	-.128	-.070	-.136	.180	-.015	.056	.118	.236*	-.102	.207*	-.196*	-			
19. SWLS	.231*	.131	.007	.029	-.111	.199*	.199*	.165	-.528*	-.192**	.176	-.084	-.211**	-.257**	-.035	-.504**	.013	-.034	-		
20. SRLE	-.066	-.158	.036	-.064	.026	.002	.020	-.029	.513**	.336*	.021	.224*	.419**	.416*	-.268**	.460**	.059	.165	-.404**	-	
21. DOI	.035	.075	.011	-.111	.114	.032	-.006	.049	.318**	-.402**	.191*	-.239*	-.266**	-.164	.283**	-.262**	.117	-.109	.289**	-.419**	-

*Note*. The presented significances are for Pearson’s correlation and two-tailed tests of significance.

RTF = Resistance to Framing, ADR = Applying Decision Rules, CRP = Consistency in Risk Perception, RSC = Resistance to Sunk Costs, RSN = Recognizing Social Norms, SMS = Self-Monitoring Scale, TEIQue-SF = Trait Emotional Intelligence Questionnaire—Short Form, TS = Time-Style, SWLS = Satisfaction With Life Scale, SRLE = Survey of Recent Life Experience, DOI = Decision Outcome Inventory.

* *p* < .05.

** *p* < .01.

In contrast, confirming the results of Study 2, significant relationships were found between the measures of social orientation and the indicators of real-life decision-making success. Especially, higher TEI was related to reports of being more satisfied with life, experiencing fewer everyday difficulties (SRLE), and being able to avoid negative outcomes associated with real-life decision making (DOI).

With respect to the relationships between the measures of time-approach and the different indicators of decision-making success, the results confirmed that approach to time is an important individual difference variable. Looking at the relationship between measures of time-approach and the DOI, procrastination behaviour had the strongest (negative) association. Moreover, relationships between the measures of time-approach and the other indicators of decision success were in line with, and confirmed, those found in Study 2.

#### Multiple regression models

As in Study 2, hierarchical multiple regression analyses were performed to investigate the predictive relationship between the different decision-making competencies and measures of real-life decision-making outcome (Table 7). As in Study 2, separate analyses were performed for the respective outcome measures of *SWLS*, *SRLE*, and *DOI*, by use of the respective blocks of predictors: 1) *gender* and *age*, 2) *DMC* and, 3a) *Social skills* and 3b) *Time-approach* respectively. That is, in line with the analyses performed in Study 2, the predictive validity of Social skills and Time-approach were tested in two separate blocks 3 of the regression model.

**Table 7:** Hierarchical regression of SWLS, SRLE, and DOI—Study 3 (*N* = 111).

	Total *R* ^2^	Adjusted *R* ^2^	Δ*R* ^2^	Test of Δ*R* ^2^
SWLS				
- Step 1: Gender and age	.02	-.00	.02	*F*(2, 106) = .92, *p* = .402
- Step 2: DMC	.08	.02	.07	*F*(5, 101) = 1.49, *p* = .198
- Step 3^a^: Social skills	.36	.30	.27	*F*(3, 96) = 13.66, *p* < .001
- Step 3^b^: Time-approach	.37	.27	.28	*F*(7, 94) = 6.01, *p* < .001
SRLE				
- Step 1: Gender and age	.02	.01	.02	*F*(2, 106) = 1.28, *p* = .282
- Step 2: DMC	.07	.01	.05	*F*(5, 101) = .98, *p* = .429
- Step 3^a^: Social skills	.37	.30	.30	*F*(3, 96) = 15.15, *p* < .001
- Step 3^b^: Time-approach	.47	.39	.40	*F*(7, 94) = 10.20, *p* < .001
DOI				
- Step 1: Gender and age	.01	-.01	.01	*F*(2, 105) = .37, *p* = .694
- Step 2: DMC	.05	-.01	.05	*F*(5, 100) = 1.00, *p* = .419
- Step 3^a^: Social skills	.24	.16	.17	*F*(3, 95) = 7.22, *p* < .001
- Step 3^b^: Time-approach	.29	.18	.24	*F*(7, 93) = 4.39, *p* < .001

*Note*. SWLS = Satisfaction With Life Scale; SRLE = Survey of Recent Life Experience; DOI = Decision

Outcome Inventory, DMC = Adult Decision-Making Competence.

^a, b^ Separate blocks in Step 3

***Predicting satisfaction with life:*** As Table 7 shows, Step 1 (*gender* and *age*) explained 2% of the variability in SWLS (n.s.). In Step 2, the *DMC* added 7% to the explained variance (n.s.). Although the contribution of the DMC block was not significant, the component *Resistance to Framing* (RTF) was found to be a significant predictor (*β* = .227, *p* = .026). Next, when the block *Social skills* was added in Step 3, the explained variance increased by 27% (*p* < .001). Here, the *TEIQue-SF* (*β* = .577, *p* < .001) was the significant predictor. When instead measures of *Time-approach* were inserted into Step 3, this block accounted for an additional 28% of the variance (*p* < .001). Here, the *anxious* (*β* = -.423, *p* < .001) and the *preference for economic time* (*β* = .228, *p* = .028) time styles were the significant predictors.

***Predicting recent life experiences:*** Using daily hassles (SRLE) as the outcome measure, multiple regression analyses showed that neither the contribution of Step 1 (*gender* and *age*) nor Step 2 (*DMC*) was significant. In Step 3, when *Social skills* was added to the model, the explained variance increased by 30% (*p* < .001). Here, the SMS subscale *ability to modify self-presentation* (*β* = .267, p = .016) and *TEIQue-SF* (*β* = -.659, *p* < .001) were the significant predictors. Moreover, using measures of *Time-approach* in Step 3 added a total of 40% of explained variance (*p* < .001). In this block, the *future-oriented* (*β* = .246, *p* = .014), *submissive* (*β* = -.235, *p* = .015), and *anxious* (*β* = .263, *p* = .006) time styles were found to be the significant predictors.

***Predicting objective decision outcome:*** When scores on the Decision Outcome Inventory (DOI) were used as the outcome measure, Step 1 (*gender* and *age*) explained 2% of the variability whereas Step 2 (*DMC*) added another 5%. However, neither Step 1 nor Step 2 made a significant contribution. Next, when measures of *Social skills* were used in Step 3 of the model, the explained variance increased by 17% (*p* < .001). In this block, the significant predictors were the SMS subscale *ability to modify self-presentation* (*β* = -.305, *p* = .013) and the *TEIQue-SF* (*β* = .475, *p* < .001). Finally, when instead measures of *Time-approach* were inserted in Step 3, this change added a total of 24% of explained variance (*p* < .001). However, although the contribution of the Time-approach block was significant, no single predictor was significant.

### Discussion

In brief, the results of Study 3 confirmed those of both Study 1 and Study 2. For example, the non-significant contribution of the DMC to the subjective decision-making outcome measures SWLS and SRLE was replicated. In addition, the non-significance of the DMC was also observed when the DOI was used as the outcome measure. This non-significant relationship was unexpected, considering the results reported by previous research [2]. In sum, the result of Study 3 supported the assumption of the present research, proposing that both social skills and aspects of time-approach are important individual difference factors to consider in a wider definition of decision-making competence. Not only was this predictive relationship replicated for subjective criteria of real-life decision-making outcomes, measured in terms of daily hassles and general satisfaction with life, but it also expanded to objective criteria as measured by the DOI.

On the one hand, relationships between the subjective outcome measures SRLE and SWLS in Study 3 were similar to those found in Study 1 and Study 2. Furthermore, the relationship between SRLE and the DOI found in Study 1 was replicated and even proved to be somewhat stronger in the professional sample for Study 3. Interestingly, in the Study 3 sample, a significant and positive relationship was found between SWLS and the DOI. This result shows that objective decision-making outcomes and subjective outcome in terms of general satisfaction can be related in some contexts.

## General Discussion

Based on the insights provided by previous research on decision-making competence [1–6] and considering the complex issue of defining decision quality [7–12], the present research explored three questions. *First*, we explored how an objective definition of real-life decision-making outcome (as instantiated by the Decision Outcome Inventory; DOI [2]) relates to subjective definitions of real-life decision-making outcome (i.e. experiences of daily hassles and satisfaction with life). The results from both a community sample (Study 1) and a professional sample (Study 3) demonstrated that individuals who had avoided objectively defined negative decision-making outcomes also reported to experience less minor difficulties in their lives. In the community sample of Study 1, no significant relationships between objective decision-making outcomes (i.e. the DOI) and subjective levels of satisfaction with life (i.e. the SWLS) were observed. Yet in the professional sample of Study 3, a significant relationship was found. The inconsistent relationship between the DOI and the SWLS does not allow a clear understanding of the relationship between objective and subjective decision-making outcomes. Nevertheless, since the results were inconsistent, the fatalistic view of a non-relationship between individuals’ decision-making and satisfaction with life, reported by previous research [62–63] is somewhat put to question. At the very least, the results of the present study indicate that more research into this relationship is called for.

*Secondly*, we explored if the previously reported relation between DMC-performance and (an objective indicator of) real-life decision-making outcomes [2] replicate when different criteria (i.e., subjective indicators) are used. The results of Study 2 and Study 3 did not support the predictive power of the DMC for other (subjective) criteria of real-life decision-making outcome. Moreover, the previously reported relationship between DMC-performance and an objective indicator (i.e., the DOI) was not replicated (Study 3).

*Third*, we explored if individual differences in decision-related a) social skills and b) time-approach add to the explanation of variance in real-life decision-making outcome(s) beyond the variance explained by the DMC. The results of Study 2 and Study 3 provides support for the idea that the existing, cognitively oriented, definition and measurement of individual differences in decision-making competence beneficially can be expanded by incorporating decision-related social skills and time-approach. Thus, social skills and time-approach contributed to the explanation for both subjective and objective criteria of real-life decision-making outcome. These results are next discussed in the contexts of decision-making competence and the definition of decision-making quality.

### Defining decision-making competence

What does it mean to be a competent decision maker? Based on the results reported in the present research the answer to this question is that it depends on which aspect(s) of decision making that is referenced. That is, if decision-making competence refers to the ability to carry out decision processes and make decisions that ultimately lead to reduced subjective experiences of everyday difficulties, higher satisfaction with life, and avoidance of objectively negative real-life decision-making outcomes, the present research provides empirical support for the suggestion that decision-related social skills and time-approach should be included in the definition of decision-making competence.

An unexpected finding in the present research was that the predictive power and benefits of the DMC measure were not replicated. DMC-performance has been suggested to encompass essential decision-making skills (see e.g., [2, 71]), i.e., decision-making skills needed in order to cope with everyday decision-making demands [1]. But our results do not provide any support for this claim. Instead, our findings indicate that the cognitively oriented definition of decision-making competence could be usefully complemented by decision-related social skills and time-approach. Yet, it is important to underline that our results do not question or diminish the benefits associated between DMC performance and different specific outcomes reported in previous research [1–6]. At the same time, our results do however point to some limitations in the usefulness and predictive validity of the DMC. For example, our results suggest that, in high-performing groups, cognitive decision-making skills may not be that important in order to understand the variance in decision-making success. However, DMC may be a useful predictor in groups with a more diverse performance [3].

Furthermore, it has been suggested that performance on the DMC components *Recognizing Social Norms* and *Resistance to Sunk Costs* relies on social skills [2, 4] or, at least, is dependent on the social aspect of the decision context [72]. In this regard, it is interesting to note that no significant correlations were found between these DMC components and the included measures of social skills. Future research should explore these issues further in order to improve our understanding of the relationships.

Previous research [25] has noted that a competent decision maker should consider the *emotional effects* (i.e. “leakage”) associated with decision biases (e.g., sunk-cost effects, framing effects), since these effects impact the experience of the decision. Consequently, although ability to resist biases in one’s judgements and decisions (e.g., DMC performance) has been found to be quite stable [2, 73], such ability may not necessarily affect subjective evaluations of decisions. For instance, the present research found that the ability to resist biases in one’s judgments and decisions (i.e., DMC-performance) was not related to subjective experiences of real-life decision-making outcomes. One interpretation of this could be that the emotional effects of leakage are fairly prevalent. However, even if it might be sensible to adhere to such leakage on some occasions, recurrent and continuous adherence would probably result in negative consequences [26]. Nevertheless, although lack of ability to resist biases may result in poor decisions, the present research suggest that what may matter more for subjective evaluations of everyday decision making is how the decision is evaluated in retrospect (e.g. in terms of accountability to both oneself and others [9, 24–25, 27].

Yet the present research did not specifically attend to leakage and thus cannot provide an answer to the rationale or negativity of adhering to leakage. However, this issue should be further explored in future research.

### Defining real-life decision-making outcome/decision quality

As we argue, a definition of decision-making competence is dependent on the criteria of decision quality. However, as illustrated by the selective review provided in the Introduction, decision quality is a problematic and debated issue [7–12, 18–20]. Therefore, we first explored this issue. In brief, one way to evaluate decision quality is to consider objective evaluations of outcomes. Such objective evaluations assess decision quality in terms of the correspondence between decision-making and normative standards [8]. However, another way to evaluate decision quality is to consider how decisions are experienced to comply with decision-makers’ goals and overall life. Such subjective evaluations assess decision quality in terms of the correspondence between decision-making and personal standards [23–25]. Although we found significant correlations between the different measures of decision quality, the moderate level of these correlations suggests that the different outcome measures to a large extent capture different aspects of quality. This indicates the usefulness of using not just one, but multiple measures in research that investigate decision quality.

### Limitations

Some limitations of the present research should be noted. For example, the design was cross-sectional and used self-reports. Nevertheless, future research should use a longitudinal design and data collection by peer-ratings and performance measures, if and when appropriate, e.g. for social skills (however, regarding the usefulness of peer-ratings see [6, 74]. Furthermore, the present research explored individual differences. In this regard the present samples could be regarded as rather small and in the case of the professional samples (Studies 2 and 3) as somewhat homogeneous. However, although similar sample sizes of somewhat homogeneous groups have been used in previous decision-making competence research [1, 5], future research should attempt to use larger samples. In Study 2, data was collected by the use of web-based and paper-and-pen questionnaires. Yet it has been demonstrated that the use of web and/or paper-and-pen does not have an effect on people’s responses [75]. The data-collection in Study 2 Moreover, the fact that no corrections of p-values where performed for the multiple statistical tests in the correlations may also be considered a limitation. However, the benefits of corrections like Bonferroni have been questioned [76], and the present research sought to respond to this issue by following the alternative recommendation to conduct repeated studies in order to explore if the main relationships were replicated.

### Concluding remarks

The results of the three studies in the present research suggest that it would be beneficial and relevant to use *different definition*s when approaching and measuring decision quality in people’s everyday lives. Arguably, one single composite outcome measure is not sufficient in order to capture the full complexity of everyday decision quality [12]. By attending to various criteria, a better understanding of decision quality and the predictive validity of decision-making competence could be attained. Future research should also investigate if the support for the expanded definition of decision-making competence, reported in the present research, replicates in other samples *and* for other criteria of decision quality. In brief, the present research has suggested and provided empirical support for the idea that the definition of decision-making competence could beneficially be extended to include decision-related social skills and time-approach. However, future research should also investigate the usefulness of other decision-related factors than the ones investigated in the present research.

## Supporting Information

S1 Dataset (SAV)

S2 Dataset (SAV)

S3 Dataset (SAV)
